# Trimester-Specific Reference Intervals of Thyroid Function Testing in Pregnant Women from Basrah, Iraq Using Electrochemiluminescent Immunoassay

**DOI:** 10.3390/diseases4020020

**Published:** 2016-04-26

**Authors:** Ammar Mohammed Saeed Almomin, Abbas Ali Mansour, Maysoon Sharief

**Affiliations:** 1 Al-Faiha Specialized Diabetes, Endocrine, and Metabolism Center (FDEMC), Basrah 61013, Iraq; ammar.almomin75@gmail.com; 2 Diabetes, Endocrine and Metabolism Division, Department of Medicine, Basrah College of Medicine, Basrah 61013, Iraq; 3 Department of Gynecology and Obstetrics, Basrah College of Medicine, Basrah 61013, Iraq; maysoonsharief60@yahoo.com

**Keywords:** thyroid function testing, reference intervals, pregnancy, electrochemiluminescent immunoassay

## Abstract

Background: Thyroid function test results of healthy pregnant women differ from those of healthy non-pregnant women. This study aimed to determine trimester-specific reference ranges for total tetraiodothyronin (T4), free T4, total triiodothyronin (T3) and thyroid stimulation hormone (TSH) using electrochemiluminescence techniques from apparently healthy pregnant women in Basrah. Material and Methods: A cross sectional study was conducted between January 2014 and June 2015. The total enrolled pregnant women were 893. Clinical examination, estimation of free T4, total T4, total T3, TSH, and anti-thyroid peroxidase (anti-TPO) using electrochemiluminescence technique done for each. Results: Trimester specific normal range of TSH in μIU/mL was 0.04–3.77, 0.30–3.21 and 0.60–4.50 μIU/mL respectively, for each trimester. For FreeT4, the trimester specific reference range was 0.8–1.53, 0.7–1.20 and 0.7–1.20 ng/dL for each trimester, respectively. The reference range for total T4 for the first, second and third trimester was 7.31–15.00, 8.92–17.38, and 7.98–17.70 μg/dL, respectively. Furthermore, last trimester specific reference range for total T3 was 0.90–2.51, 1.99–2.87 and 1.20–2.70 ng/mL, respectively. Conclusion: Using this thyroid function study, we established for first time trimester-specific reference ranges for each thyroid function test and thyroid antibody status for the first time in Iraq. The reference ranges are different from all previous studies outside Iraq and the reference kit range from the method we used.

## 1. Introduction

Diseases of the thyroid gland are common, affecting about 5% of the general population, and predominantly affect females [1]. Thyroid gland dysfunction is relatively common during pregnancy. The prevalence of hyperthyroidism is approximately 0.4%, subclinical hyperthyroidism about 3.3% [2], hypothyroidism about 0.3% [3], and subclinical hypothyroidism may reach 2.5% or more [4].

Dietary adequacy is different among different geographical areas in the world. The World Health Organization (WHO) estimates that two billion people are iodine-deficient [5], and hypothyroidism due to iodine deficiency can occur at any time in life, but the most critical period is when it occurs during fetal development and early childhood. When occurring early in pregnancy, hypothyroidism and the development of thyroid autoantibodies during pregnancy associated with maternal morbidity later in life [6]. Treated maternal hypothyroidism is not associated with adverse perinatal outcome [7].

The significance of detecting maternal thyroid abnormalities during pregnancy is that hypothyroidism may be associated with miscarriages, low birth weight, anemia, pregnancy-induced hypertension, preeclampsia, abruption placenta, postpartum hemorrhage, congenital circulation defects, fetal distress, preterm delivery, and poor vision development, in addition to the probable neuropsychological defect in the child [8,9,10].

Current guidelines advocate thyroid stimulation hormone (TSH) as a nearly universal thyroid screening test in non-hospitalized patients with intact hypothalamic/pituitary function [11]. In addition, thyroid hormones should also be measured to clarify the picture of thyroid dysfunction when indicated.

Currently, total tetraiodothyronin(T4) is preferred over free T4 in the pregnancy period after adjustment by a factor of 1.5 to compensate for the expected thyroxin-binding globulin (TBG) elevation [10]. Thyroid function test results of healthy pregnant women differ from those of healthy non-pregnant women. This calls for pregnancy-specific and ideally trimester-specific reference intervals for all thyroid function tests, but in particular for the most widely applied tests, TSH, free T4, total T4, and total triiodothyronin (T3) [12].

Studies have shown that hypothyroxinemia during pregnancy is associated with adverse outcome, and women with thyroid autoimmunity are at increased risk of pregnancy complications, thus thyroid function assessment in anti-thyroid peroxidase antibodies(anti-TPO) positive pregnant women seems beneficial [13,14]

Although screening for thyroid dysfunction in healthy non-pregnant woman is not recommended, thyroid screening in pregnancy is controversial. Some suggest targeted screening (case finding) of only the high-risk group, while others recommend TSH screening for all pregnant women by the ninth week of gestation or at the time of their first visit [15,16].

### Objective

To determine trimester-specific reference ranges for total T4, free T4, total T3 and TSH using electrochemiluminescence (ECL) technique from apparently healthy pregnant women in Basrah.

## 2. Material and Methods

### 2.1. Setting

This study was done between January 2014 and June 2015. Participants for this cross-sectional study were apparently healthy pregnant women selected from women attending primary health centers (PHC), hospitals, out-patient clinic, and private gynecologic clinics in the city of Basrah (southern Iraq).

Instructions were given to the primary care doctors and specialists to refer pregnant women attending routine antenatal visits to assess their thyroid status at the Al-Faiha Specialized Diabetes, Endocrine, and Metabolism Center (FDEMC).

This study involved 893 selectively referred pregnant women; 31 of them were further excluded after detailed history and physical examination, and 9 women were also excluded because of high TSH value >10 μIU/mL. A total of 643 women were tested for anti-TPO; of them, only 103 patient tested positive, while the remaining 540 women distributed throughout different trimesters of pregnancy, 123 women in the first trimester, 246 women in the second trimester, and the remaining 171 women in the third trimester.

After ensuring patient acceptance to participate in the study, 3–5 mL venous blood samples were taken using a tube that contained clot activator from the pregnant women, and then centrifuged to be assayed on the same day. In addition to patient sampling, pregnant women were also subjected to physical examination and brief questionnaire including some parameters useful in the study. All information taken from the pregnant women have been stored in computerized systems at FDEMC.

Each patient included in the study was sampled once during her pregnancy and was not sampled again throughout the same pregnancy; this is to avoid missing patient follow-up, to enlarge sample size, and to increase sample variety.

### 2.2. Subjects

**Figure 1:**
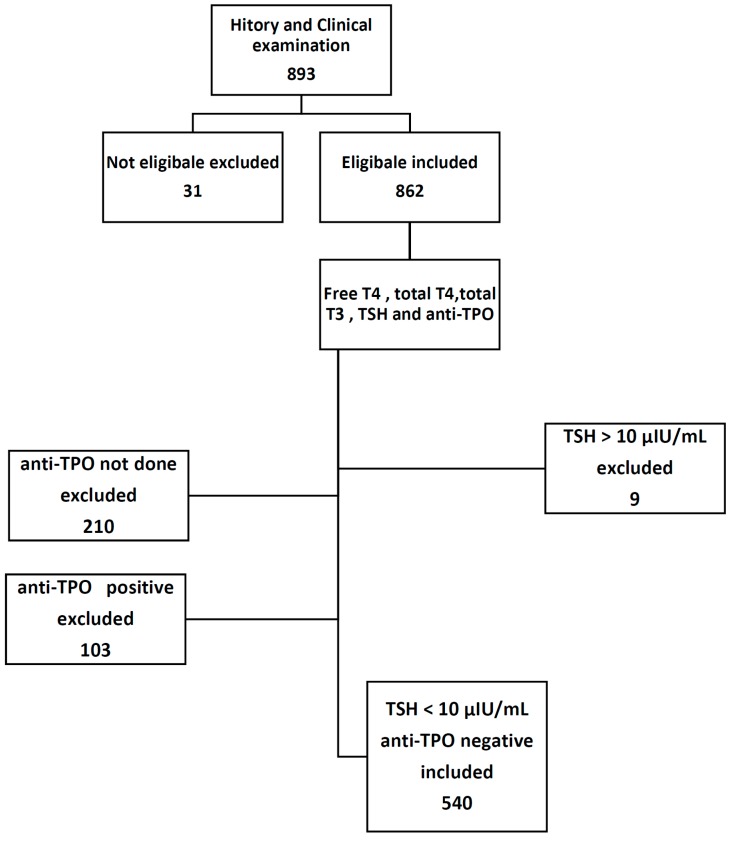
Study protocol.

Subject inclusion criteria (Figure 1) are apparently healthy pregnant women with uncomplicated single intrauterine gestations. The first trimester was considered from Weeks 1–12, second from Weeks 13–27, and Weeks 28–40 constituted the third trimester. All subjects provided verbal informed consent, and the research protocol was approved by the University of Basrah.

### 2.3. Exclusion Criteria

Women with the following criteria were excluded:

- History of hyperemesis gravidarum, thyroid illness or use of medication known to affect thyroid function like amiodarone, lithium, steroids, and non-steroidal anti-inflammatory drugs.
- Twin pregnancy.
- The family history of thyroid illness.
- The presence of more than mild goiter on clinical ground.
- Overt hypothyroidism or hyperthyroidism.
- Women with significant acute or chronic diseases were also excluded, leaving only healthy or apparently healthy pregnant women to participate in the study.
- TSH >10 (nine pregnant women excluded).
- Anti-TPO positive pregnant women.

### 2.4. Main Outcome Measure

The main outcomes measured include clinical examination, free T4, total T4, total T3, TSH, and anti-TPO using ECL technique done for each pregnant woman.

### 2.5. Biochemical Tests

All biochemical tests were completed using an ECL technique with commercially available kits from Roche Diagnostics (Germany) with cobas e 411 analyzer. The hormonal analysis was done in the FDEMC.

### 2.6. Research Instruments

#### Cobas e 411 Analyzer

Roche Electro-ChemiLuminescence Immuno Assays, “ECLIA,” Elecsys TSH test is a 3rd generation TSH test.

TSH measuring range 0.005–100.0 μIU/mL with a reference range of 0.27–4.2 μIU/mL and specified the intra-assay precision of 0.1–4 μU/mL (<5% CV).

Total T4 measuring range 0.420–24.86 μg/dL with reference range 5.1–14.1 μg/dL and specified the intra-assay precision of <50 nmol/L (<5% CV).

FreeT4 measuring range 0.023–7.77 ng/dL with reference range 0.93–1.7 ng/dL and specified the intra-assay precision of 25–100 pmol/L (<3% CV).

Total T3 measuring range 0.195–6.51 ng/mL with reference range 0.8–2.0 ng/mL and specified the intra-assay precision of 2.5–10 nmol/L (<3% CV).

Anti-TPO measuring range 5–600 IU/mL with positive values >34 IU/mL and specified the intra-assay precision of >40 IU/ML (<7% CV).

### 2.7. Statistical Analysis

For statistics, all data were computed and analyzed using SPSS, (version 15.0, SPSS Inc., Chicago, IL, USA). Continuous variables were summarized as the mean (± standard deviation (SD), and categorical variables were summarized as a percentage. The range of 5th to 95th centile was considered the reference range for each test separately. One-Way analysis of variance (one-way ANOVA) was used to compare mean.

## 3. Results

The mean age for the total pregnant women was 27.9 ± 7.3 years with total age range of 14–48. There were 123 women (22.8%) in the first trimester, 246 (45.5%) in the second trimester and 171 (31.7%) in the third.

Table 1 shows the age distribution of the women who participated in this study, where the most frequent group is between the ages of 20–30 years (242, 45.1%), followed by the age group of 30–40 years (181, 33.6%).

Table 2 shows the thyroid function test parameters for each trimester, where TSH value increased with advancing pregnancy from 1.51 ± 1.16 μIU/mL in the first trimester to 1.87 ± 1.11 μIU/mL in the third trimester, and the difference was statistically significant. The free T4 level decreased from 1.15 ± 0.23 ng/dL in the first trimester to 0.90 ± 0.16 ng/dL in the third trimester. Total T4 and total T3 both increased during pregnancy where total T4 is 11.07 ± 2.62 μg/dL in the first trimester and 12.43 ± 3.0 μg/dL in the third trimester, while the total T3 level is 1.62 ± 0.47 μg/dL and 1.99 ± 0.44 μg/dL in the first and third trimester, respectively. All three of these thyroid function differences between trimesters are statistically different.

**Figure 2:**
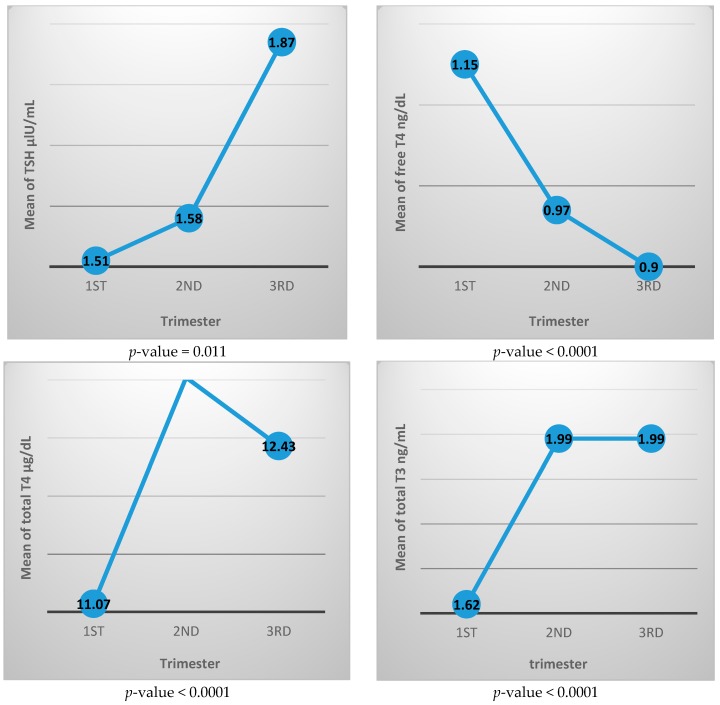
Mean value of thyroid function tests for each trimester.

Figure 2 shows the mean value of each thyroid function test during different trimesters. It generally illustrates increasing level of TSH, total T3, and total T4 with advancing pregnancy, while free T4 value is decreasing with pregnancy progression.

Table 3 shows the reference range for each of the thyroid test from 5th centile to 95th centile for each trimester. Trimester specific normal ranges of TSH in μIU/mL were 0.04–3.77, 0.30–3.21 and 0.6–4.5 μIU/mL, respectively, for first, second and third trimesters. For Free T4, the trimester specific reference ranges were 0.8–1.53, 0.7–1.20 and 0.7–1.20 ng/dL for first, second and third trimesters, respectively. The reference ranges for total T4 for the first, second and third trimesters were 7.31–15.0, 8.92–17.38, and 7.98–17.70 μg/dL, respectively. Finally, trimester specific reference ranges for total T3 were 0.90–2.51, 1.30–2.87 and 1.2–2.70 ng/mL, sequentially.

Table 4 shows the thyroid autoimmunity detected by positive anti-TPO. In this table from 853 women included in the initial evaluation, anti-TPO testing was done in only 643 women, 103 of them (16.2%) were tested positive.

## 4. Discussion

There are many trimester specific reference ranges studies among the literature (Table 5). However, the comparison between the studies is not an easy task mostly due to using different laboratory methods for estimation of thyroid hormones, and also due to different inclusion and exclusion criteria. Some use 2.5th–95th centile range while others use 5th–95th centiles. Furthermore, some used mean ± SD reference.

This study includes 540 pregnant women divided into three trimesters. The age distribution of persons involves pregnant women at both extremes of reproductive age, including about 14% below 20 years of age, down to 14 years of age and about 7% above 40 years of age up to 48 years old. The mean age of women in the study is about 28 years old. Approximate to these age limits are also seen in larger study in Spain, where 1198 pregnant women between the age of 15–45 years were targeted by the study [26]. 

Although, optimally, participant distribution should be equal in each trimester, we could not maintain equality among trimesters, but comparable pregnants number is maintained.

Several physiological changes occur during pregnancy, which in turn may affect the normal values of the most widely used thyroid function test parameters. Several studies of the thyroid function tests among different geographical areas have been done with different values of the normal reference range, suggesting that nearly every population may have unique normal values [27,28].

The Large variation in normal values of thyroid function is a real problem all over the world due to assay specific methods, ethnic variations, and difference in body mass index. Thus every institution should have its own reference interval of normal thyroid function in pregnancy because missing mild thyroid dysfunction in pregnancy can have grave effects on the fetus and mothers [29].

In this study, we target population with special dietary habits, special environmental and social circumstances and special level of education and health services.

In this study, by tracking the TSH mean value for each trimester (Figure 2), we noticed that the TSH value in the first trimester is lower than in the second trimester while the third trimester has the highest value during pregnancy. 

The initial decrease in TSH value is likely due to human chorionic gonadotrophine with its TSH mimetic effect that is characteristic in early pregnancy. With fading up of human chorionic gonadotrophine effect with advancing pregnancy, the TSH concentration starts to rise to reach its highest concentration in late pregnancy. This upward sloping curve in the TSH level was also observed by Glinoer *et al.* in 1990, as well as by other studies done in Malaysia and China [17,25,30].

The lower limit of the reference range of TSH in this study was lower in the first trimester than that of the non-pregnant women ranging from 0.04 μIU/mL compared to 0.27 μIU/mL for non-pregnant women using ECL technique from Roche Diagnostics using cobas e 411 analyzers. In similar studies, on Indian pregnant women using similar analytic methods, the TSH values usually had higher normal limits compared to our results [18], while a study is done in China showed comparable results to ours, especially in early pregnancy [24].

The reference range of TSH in the second trimester was narrower than those in the first and third trimesters. The ATA suggests that trimester-specific TSH values should be used in every population. When trimester-specific reference intervals are not available, the following reference intervals can be used: first trimester, 0.1–2.5 mIU/L; second trimester, 0.2–3.0 mIU/L; and third trimester, 0.3–3.0 mIU/L [10].

The results of the second-trimester reference range are closer to the values suggested by the ATA for pregnant women than the TSH range in the first and third trimesters. 

In general, the TSH values gained from the current study differ from that suggested by the ATA for pregnant women, *i.e.* the trimester-specific reference range universally suggested by the ATA may be not applicable to our society. 

Levels of free T4 have been reported to decline throughout pregnancy [21]. In this study, the mean value of free T4 shows progressive decline from the first trimester to the third trimester. This is an expected finding where the increased binding of the thyroid hormones to the increasingly produced TBG makes the free form to be reduced with advancing pregnancy. 

The lack of standardizations of free thyroid hormone measurements makes it difficult to apply a universally accepted standard reference value for it. Thus, it was necessary to develop our own normal values.

The normal reference range of free T4 in this study is persistently lower than that suggested for the general population throughout all stages of pregnancy where the highest range in the first trimester is 0.8–1.53 ng/dL compared to the normal population range 0.93–1.7 ng/dL. This finding is consistent with a study from Korea [23].

It is easier to measure the total thyroid hormone concentrations that are measured at nanomolar levels than direct serum freeT4 and free T3 that circulate in the picomolar range. On the contrary, to the free thyroid hormones, which lack international standardization, the total thyroid hormone has been well standardized for more than 40 years, and pregnancy effect on the normal range of total thyroid hormone can be overcome by multiplying the hormone concentration by 1.5 to compensate for the expected increase in the total thyroid hormone level [10]. The total T4 and total T3 values seen in this study approximately were 7.3–15 μg/dL and 0.9–2.5 μg/dL, respectively, in the first trimester, and 7.9–17.7μg/dL and 1.20–2.7 μg/dL, respectively, in the third trimester.

The values of both total T3 and total T4 increase from the first trimester to the second trimester, and then this increase nearly plateaus, with minimal reduction at the end of pregnancy. 

This could be due to the increased binding effect of TBG, which tends to increase with advancing pregnancy. Very similar findings were seen in an Iranian study carried out about ten years ago and by an American study conducted between 1988 and 1994 [20,31].

In this study, 16.2% of women tested positive for anti-TPO (Table 4). This implies a significant number of women had thyroid autoimmunity. This is a well established fact, were 10%–20% of apparently normal pregnant women were found to be anti-TPO positive [32].

Using this study, we established the normal values of thyroid function test in pregnancy for the first time in Iraq. The kits trimester-specific normal values are different from values obtained in this study. For TSH, the kits trimester-specific reference range were 0.33–4.59, 0.35–4.1, and 0.21–3.15 μIU/mL; for free T4 they were 0.94–1.52, 0.75–1.32 ng/dL and 0.65–1.21 ng/dL; for total T4 they were 7.33–14.8, 7.93–16.1 and 6.95–17.7 μg/dL; and for total T3 they were 1.05–2.30, 1.29–2.62, and 1.35–2.62 ng/mL, respectively, for the first, second, and third trimesters [33]. This signifies the importance of developing local reference ranges for our population as it differs from the kit reference range recommended during pregnancy. 

Iran data on a trimester-specific reference ranges for TSH were 0.2–3.9, 0.5–4.1 and 0.6–4.1 μIU/mL, and for total T4 were 8.2–18.5, 10.1–20.6, and 9.0–19.4 μg/dL for first, second, and third trimesters, respectively. Unlike our study, the Iranian study excluded thyroglobulin antibody positive and iodine deficient pregnant women. It also included only Persian ethnicity and depends on following the same sample of women during each trimester [20]. While in India, the TSH reference interval for first, second and third trimester were 0.25–3.35, 0.78–4.96 and 0.9–4.6 μIU/mL, respectively, while for free T4, they were 0.64–2.0, 0.53–2.02 and 0.64–1.99 ng/dL. The Indian study differs from ours as it used the ELISA technique for estimation of thyroid related hormones and excluded pregnant women with previous spontaneous abortion [19].

## 5. Conclusion

This thyroid function study established trimester-specific reference ranges for each thyroid function test and thyroid antibody status for the first time in Iraq. The reference ranges are different from all previous studies outside Iraq and the reference kit ranges from the method we used.

## Figures and Tables

**Table 1:** Age distribution of pregnant women.

Age in Years	Number (%)
<20	76 (13.9)
20–30	242 (45.2)
30–40	181 (33.6)
>40	41 (7.3)
Total	540 (100)

**Table 2:** The mean values of thyroid function test parameters per each trimester.

Trimester	TSH μIU/mL Mean ± SD	*p* Value	FreeT4 ng/dL Mean ± SD	*p* Value	TotalT4 μg/dL Mean ± SD	*p* Value	TotalT3 ng/mL Mean ± SD	*p* Value
First	1.51 ± 1.16	0.011	1.15 ± 0.23	<0.0001	11.07 ± 2.62	<0.0001	1.62 ± 0.47	<0.0001
Second	1.58 ± 0.94		0.97 ± 0.16		13.02 ± 2.59		1.99 ± 0.47	
Third	1.87 ± 1.11		0.90 ± 0.16		12.43 ± 3.0		1.99 ± 0.44	

**Table 3:** The 5th and 95th centile values of thyroid function tests parameters per each trimester.

Tests	Trimester	5th Centile	95th Centile
TSH μIU/mL	First trimester	0.04	3.77
	Second trimester	0.30	3.21
	Third trimester	0.60	4.50
FreeT4 ng/dL	First trimester	0.80	1.53
	Second trimester	0.70	1.20
	Third trimester	0.70	1.20
Total T4 μg/dL	First trimester	7.31	15.0
	Second trimester	8.92	17.38
	Third trimester	7.98	17.70
Total T3 ng/mL	First trimester	0.90	2.51
	Second trimester	1.30	2.87
	Third trimester	1.20	2.70

**Table 4:** Thyroid peroxidase antibody (anti-TPO) positivity.

Anti-TPO Level IU/mL	Number (%)
Missed values	210 (24.6)
0–34	540 (63.3)
>34	103 (12.1)
Total	853 (100)

**Table 5:** Comparison between different countries in the Trimester specific reference interval.

Country, Year	Thyroid Test	Sample Size	First Trimester	Second Trimester	Third Trimester	Methods/Instrument
Basrah, Iraq, 2015 5th–95th centile	TSH μIU/mL	540	0.04–3.77	0.30–3.21	0.6–4.5	ECL/*cobas e411* analyzer
Mean ± SD			1.51 ± 1.16	1.58 ± 0.94	1.87 ± 1.11	
5th–95th centile	FreeT4 ng/dL		0.8–1.53	0.70–1.20	0.70–1.20	
Mean ± SD			1.15 ± 0.23	0.97 ± 0.16	0.90 ± 0.16	
5th–95th centile	Total T4 μg/dL		7.31–15.0	8.92–17.38	7.98–17.7	
Mean ± SD			11.07 ± 2.62	13.02 ± 2.59	12.43 ± 3.0	
5th–95th centile	Total T3 ng/mL		0.90–2.51	1.30–2.87	1.20–2.70	
Mean ± SD			1.62 ± 0.47	1.99 ± 0.47	1.99 ± 0.44	
Ref. Current study						
Malaysia, 2009 Mean ± SD	TSH MIU/L	626	1.04 ± 0.08	1.82 + 0.07 mIU/L	1.92 + 0.06	Abbott AxSYM immunoassay platform.
Mean ± SD	FreeT4 pmol/L		13.86 ± 5.9	9.35 + 2.07	8.40 + 1.30	
Mean ± SD	Total T4 nmol/L		143.56 ± 38.26	140.89 + 26.99	138.03 + 22.79	
Mean ± SD	Total T3 nmol/L		1.18 ± 0.38	1.29 + 0.24	1.29 + 0.30	
Ref. [17]						
New Delhi, India, 2008 5th–95th centile	TSH μIU/mL	541	0.6–5	0.435–5.78	0.74–5.7	ECL/Elecsys 1010 analyzer
5th–95th centile	FreeT4 pmol/L		12–19.45	9.48–19.58	11.3–17.71	
Ref. [18]						
North Kolkata, West Bengal, India, 2014 Mean ± SD	TSH μIU/mL	* 402	0.25–3.35	0.78–4.96	0.9–4.6	ELISA
Mean ± SD	FreeT4 ng/dL		0.64–2.0	0.53–2.02	0.64–1.99	
Ref. [19] *						
Tehran, Iran, 2013 5th–95th centile	TSH μIU/mL	*152	0.2–3.9	0.5–4.1	0.6–4.1	Immunoenzymometric assay (IRMA) /Wizard, Wallac Oy, Turku, Finland).
5th–95th centile	Total T4 (μg/dL)		8.2–18.5	10.1–20.6	9.0–19.4	
5th–95th centile	Total T3 (ng/dL)		138–278	155–328	137–324	
Ref. [20] *						
Tabriz, Iran, 2005 Mean + SD	TSH μIU/mL	229	1.71 + 1.38	1.89 + 1.24	2.12 ± 0.77	Radio immunoassay/Gammamatic II gammacounter (Contron, Switzerland).
Mean + SD	FreeT4 pmol/L		14.90 ± 4.67	13.07 ± 3.06	6.91 + 3.20	
Mean + SD	Total T4 nmol/L		87.98 + 40.87	94.30 ± 41.70	123.80 + 50.50	
Mean + SD	TT3 nmol/L		2.54 + 1.41	3.15 + 1.76	2.90 ± 1.5	
Ref. [21]						
Australia, 2013 5th–95th centile	TSH μIU/mL	130	0.05–2.33	0.47–2.71	0.42–2.65	Beckman Dxl 800 analysers
Mean + SD	FreeT4 pmol/L		5.9–15.5	4.9–11.3	4.5–11.0	
Ref. [22]						
Korea, 2012 Mean + SD	TSH μIU/mL	531	0.01–4.10	0.01–4.26	0.15–4.57	ECL/Elecsys thyroid tests, Roche Diagnostics
Mean + SD	FreeT4 ng/dL		0.83–1.65	0.71–1.22	0.65–1.13	
Ref. [23]						
Jiangsu, China, 2010 2.5th–95th centile	TSH μIU/mL	301	0.02–3.65	0.36–3.46	0.44–5.04	Electrochemistry immunoassay (ECL)/COBAS e601
2.5th–95th centile	FreeT4 pmol/L		11.85–21.51	9.45–6.26	9.30–17.14	
Ref. [24]						
Shanghai, China, 2013 2.5th–95th centile	TSH mIU/L	2743	0.06–3.13	0.07–4.13	0.15–5.02	Beckman Coulter UniCel™ DxI 600.
2.5th–95th centile	FreeT4 pmol/L		8.72–15.22	7.10–13.55	6.16–12.03	
Ref. [25]						

*: Excluded Anti-TPO + ve.
